# Improving mental health and psychosocial wellbeing in humanitarian settings: reflections on research funded through R2HC

**DOI:** 10.1186/s13031-020-00317-6

**Published:** 2020-10-30

**Authors:** Wietse A. Tol, Alastair Ager, Cecile Bizouerne, Richard Bryant, Rabih El Chammay, Robert Colebunders, Claudia García-Moreno, Syed Usman Hamdani, Leah E. James, Stefan C.J. Jansen, Marx R. Leku, Samuel Likindikoki, Catherine Panter-Brick, Michael Pluess, Courtland Robinson, Leontien Ruttenberg, Kevin Savage, Courtney Welton-Mitchell, Brian J. Hall, Melissa Harper Shehadeh, Anne Harmer, Mark van Ommeren

**Affiliations:** 1 Section of Global Health, Department of Public Health, University of Copenhagen, Øster Farimagsgade 5, bg 9, DK-1014 Copenhagen, Denmark; 2 Peter C. Alderman Program for Global Mental Health, HealthRight International, New York, NY USA; 3 Department of Mental Health, Johns Hopkins Bloomberg School of Public Health, Baltimore, MD USA; 4 Institute for Global Health and Development, Queen Margaret University, Edinburgh, UK; 5 Mailman School of Public Health, Columbia University, New York, NY USA; 6 Mental Health, Child Care Practices, Gender and Protection, Action Contre La Faim, Paris, France; 7 School of Psychology & Traumatic Stress Clinic, University of New South Wales, Sydney, Australia; 8 National Mental Health Programme, Ministry of Public Health, Beirut, Lebanon; 9 Department of Psychiatry, Saint Joseph University, Beirut, Lebanon; 10 Global Health Institute, University of Antwerp, Antwerp, Belgium; 11 Department of Sexual and Reproductive Health and Research, World Health Organization, Geneva, Switzerland; 12 Human Development Research Foundation, Islamabad, Pakistan; 13 Institute of Behavioral Science, University of Colorado, Boulder, CA USA; 14 Center for Mental Health, College of Medicine and Health Sciences, University of Rwanda, Kigali, Rwanda; 15 HealthRight Uganda, Arua, Uganda; 16 Muhimbili University of Health and Allied Sciences, Dar Es Salaam, Tanzania; 17 Jackson Institute of Global Affairs, Yale University, New Haven, CT USA; 18 Department of Anthropology, Yale University, New Haven, CT USA; 19 Department of Biological and Experimental Psychology, Queen Mary University of London, London, UK; 20 Department of International Health, Bloomberg School of Public Health, Johns Hopkins University, London, UK; 21 International Medical Relief Services (IMRES), Prior association: Arq International, Europe, Netherlands; 22 Evidence Building, World Vision International, Geneva, Switzerland; 23 Institute of Behavioral Science and Colorado School of Public Health, University of Colorado, Boulder, Denver USA; 24 Global and Community Mental Health Research Group, New York University (Shanghai), Shanghai, People’s Republic of China; 25 Institute of Global Health, Faculty of Medicine, University of Geneva, Geneva, Switzerland; 26 Elhra, London, UK

## Abstract

Major knowledge gaps remain concerning the most effective ways to address mental health and psychosocial needs of populations affected by humanitarian crises. The Research for Health in Humanitarian Crisis (R2HC) program aims to strengthen humanitarian health practice and policy through research. As a significant portion of R2HC’s research has focused on mental health and psychosocial support interventions, the program has been interested in strengthening a community of practice in this field. Following a meeting between grantees, we set out to provide an overview of the R2HC portfolio, and draw lessons learned. In this paper, we discuss the mental health and psychosocial support-focused research projects funded by R2HC; review the implications of initial findings from this research portfolio; and highlight four remaining knowledge gaps in this field. Between 2014 and 2019, R2HC funded 18 academic-practitioner partnerships focused on mental health and psychosocial support, comprising 38% of the overall portfolio (18 of 48 projects) at a value of approximately 7.2 million GBP. All projects have focused on evaluating the impact of interventions. In line with consensus-based recommendations to consider a wide range of mental health and psychosocial needs in humanitarian settings, research projects have evaluated diverse interventions. Findings so far have both challenged and confirmed widely-held assumptions about the effectiveness of mental health and psychosocial interventions in humanitarian settings. They point to the importance of building effective, sustained, and diverse partnerships between scholars, humanitarian practitioners, and funders, to ensure long-term program improvements and appropriate evidence-informed decision making. Further research needs to fill knowledge gaps regarding how to: scale-up interventions that have been found to be effective (e.g., questions related to integration across sectors, adaptation of interventions across different contexts, and optimal care systems); address neglected mental health conditions and populations (e.g., elderly, people with disabilities, sexual minorities, people with severe, pre-existing mental disorders); build on available local resources and supports (e.g., how to build on traditional, religious healing and community-wide social support practices); and ensure equity, quality, fidelity, and sustainability for interventions in real-world contexts (e.g., answering questions about how interventions from controlled studies can be transferred to more representative humanitarian contexts).

## Background

### Mental health and psychosocial support in humanitarian settings: the role of research

Humanitarian crises, including armed conflicts and disasters (e.g., triggered by natural or man-made events), are commonly associated with substantial psychological and social suffering. The mental health and psychosocial impacts of humanitarian crises on individuals, families, and communities may be extensive yet highly diverse, ranging from quick recovery to long-term negative impacts [1]. In acknowledgement of the diversity of potential needs and local capacities in humanitarian crises, international guidelines recommend multi-layered, complementary supports that focus on goals ranging from: psychological and social considerations in provision of all humanitarian assistance to protect dignity and human rights (e.g., ensuring the active participation of affected populations, including marginalized communities, in reconstruction efforts; following cultural preferences when burying deceased individuals where possible); strengthening existing family and community support systems (e.g., training facilitators of youth clubs in emotional and social support skills; family reunification); and providing focused care for people with specific mental health and psychosocial problems (e.g., psychotherapeutic and pharmacological interventions for people with mental disorders; community-based group sessions with perpetrators of gender-based violence) [2]. To cover this broad set of goals, guidelines refer to the composite term ‘mental health and psychosocial support’ (MHPSS), defined as “any type of local or outside support that aims to protect or promote psychosocial well-being and/or prevent or treat mental disorder” [2]. Existing guidelines recommend MHPSS implementation across various humanitarian sectors, including health, protection, nutrition, camp coordination and management, education, and livelihoods [3, 4].

Research focused on MHPSS is crucial to humanitarian practice and policy in several ways. For example, research may assist in: guiding and prioritizing humanitarian programming by understanding the most critical mental health and psychosocial needs and unpacking the risk, protective, and promotive factors linked to MHPSS concerns; improving interventions by testing assumptions in MHPSS program theories of change; evaluating whether and how both locally and externally developed MHPSS activities meet their aims (e.g., efficacy); examining how proven interventions may most effectively be disseminated and implemented; strengthening needs assessments and program monitoring through the development and testing of measurement tools; and, understanding barriers and facilitators to implementing MHPSS activities [5].

### Disconnect between MHPSS research and practice

Systematic reviews have exposed tensions between MHPSS research and practice, reflecting a continued disconnect between research and humanitarian practice more broadly [6]. As is the case in other humanitarian fields [7], the most rigorously studied MHPSS interventions are not those most commonly implemented in humanitarian settings, while those most commonly implemented in humanitarian settings have received relatively little scrutiny [8–10]. This issue was highlighted by a consensus-based research agenda that consolidated inputs from MHPSS researchers and practitioners [11]: whereas published research in humanitarian settings has commonly focused on posttraumatic stress disorder (PTSD), depression, and anxiety, consensus-based research priorities have focused on broader, applied, contextual and methodological issues, such as identification of critical drivers of risk and resilience, appropriate methods for information gathering as part of MHPSS programming, the effectiveness of school and family interventions, and the integration of lived experiences and local perspectives on recovery. Although many researchers and practitioners operate in both academic and implementation settings, gaps in knowledge are exacerbated by the lack of sustained interaction between scholars and humanitarian practitioners, and respective differences in approach which may be summarized under the terms of scholarly ‘excellence’ vs practical ‘relevance’ [12].

In this paper, we describe an initiative currently underway that aims both to fill critical knowledge gaps and to better connect MHPSS research and practice. As a group of scholars, practitioners, and the research funder engaged with this effort, we summarize our collective approaches, draw lessons from initial findings, and highlight areas needing continued research investment.

## The R2HC initiative

This paper focuses on the portfolio of MHPSS research funded by Elrha’s Research for Health in Humanitarian Crises (R2HC) program, which aims to improve health outcomes by strengthening the evidence base for public health interventions in humanitarian crises. R2HC is funded by Wellcome, and the UK government’s Foreign, Commonwealth and Development Office and the National Institute for Health Research. R2HC’s funding is not specific to MHPSS, but this has emerged as a key focus of funding across several research calls. The broader funding landscape for MHPSS research includes initiatives focused on global mental health interventions (i.e., not specific to humanitarian settings, such as Grand Challenges Canada Global Mental Health), as well as funding from donors with broader humanitarian, health, humanities and social sciences, global health, and mental health mandates (see e.g. the International Alliance of Mental Health Research Funders: https://iamhrf.org/).

R2HC aims to strengthen the potential impact of research on humanitarian practice in several ways. A fundamental principle is that funded research must be conducted through academic-humanitarian partnerships to ensure relevance, academic rigor, operational feasibility and greater potential for impact [13]. Grantees are supported to develop strategic engagement and communication strategies to help achieve uptake of research findings, and are required to communicate their results in accessible formats (blogs, research snapshots, open access publications). In addition, R2HC holds regular research conferences with the aim of stimulating interaction between researchers, practitioners, policy makers, and humanitarian and research funders.

The R2HC program has supported a significant number of studies addressing MHPSS interventions. This has provided the opportunity for R2HC to collaborate with a community of practice in this field. Following a meeting between grantees in 2017, we set out to provide an overview of the R2HC MHPSS portfolio, and document what this told us about the research being funded. In writing this paper, we build on a summary of the R2HC-funded MHPSS studies that was commissioned in preparation for the above meeting (led by BH) and notes from key discussion points raised at the time. We invited further grantees (as new MHPSS research projects were funded in annual calls) to critically reflect on the content as the paper developed.

## R2HC-funded MHPSS research

Between 2014 and 2019, R2HC funded 18 academic-practitioner partnerships for MHPSS research through six annual calls for proposals (see Table 1 for an overview). MHPSS research projects comprised more than a third (18 out of 48, 38%) of the overall R2HC portfolio over this period with an approximate value of £7.2 million. The 18 funded MHPSS research projects have been implemented in 11 countries, within four of the six WHO global regions (the Western Pacific and European regions were not covered). Most projects have occurred in the African (10 projects) and Eastern Mediterranean regions (six projects). Ten have focused on refugees.

**Table 1:** Overview of mental health and psychosocial support research supported by the Research for Health in Humanitarian Crises program

Location	Topic	Design	Status	Partners
Democratic Republic of the Congo	Evaluation of a community-based intervention to reduce Gender Based Violence working with men who are perceived to be violent	Mixed methods; cluster randomized controlled trial	Ongoing	University of Rwanda, Institut Supérieur du Lac, Living Peace Institute
Haiti, Nepal	Evaluation of a community-based mental health integrated disaster preparedness intervention with natural disaster-prone communities	Randomized controlled trial (2 studies); Matched cluster comparison (1 study)	Completed	University of Colorado; Soulaje Lespri Moun (SLM, Haiti); Transcultural Psychosocial Organization Nepal (TPO Nepal)
Jordan, Nepal, Uganda	Evaluation of the longer-term mental health, developmental and systems impact of child friendly spaces (CFS) in humanitarian emergencies	Longitudinal controlled cohorts	Completed	World Vision and Columbia University in collaboration with Save the Children, Unicef, and Plan International
Jordan	Evaluation of a profound stress attunement psychosocial intervention with Syrian refugee and Jordanian adolescents	Mixed methods randomized controlled trial	Completed	Yale University; Queen Margaret University, Edinburgh; Mercy Corps; Taghyeer; University of Western Ontario; Harvard University
Jordan	Evaluation of a transdiagnostic, multi-component behavioral intervention for early adolescent Syrian refugees and their caregivers (Early Adolescent Skills for Emotions) (EASE)	Mixed methods, feasibility and fully powered cluster randomized trial	Ongoing	University of New South Wales, Noor Al Hussein Institute for Family Health
Lebanon	Adaptation and evaluation of a transdiagnostic psychotherapy for delivery by trained lay counsellors over the phone (Common Elements Treatment Approach) (CETA)	Mixed methods, pilot randomized controlled trial	Ongoing	Queen Mary University of London, Médecins du Monde, Lebanon; American University of Beirut, Lebanon; Johns Hopkins University, USA; Medical School Hamburg, Germany
Lebanon	Evaluation of the effectiveness and cost-effectiveness of Step-by-Step (SbS), delivered electronically, with Syrian refugees	Mixed methods randomized controlled trial	Ongoing	World Health Organization, International Medical Corps (IMC); VU University Amsterdam; United Nations High Commissioner for Refugees (UNHCR); AFMM & St Joseph University, Lebanon; University of Zurich
Liberia, Sierra Leone	Retrospective investigation of the deployment of psychological first aid (PFA) in the Ebola outbreaks in West Africa and, prospective examination of roll-out across the health sectors in Sierra Leone.	Mixed methods, controlled cohort	Completed	War Trauma Foundation, Queen Margaret University; Vrije Universiteit Amsterdam; University of Makeni; Liberia Center for Outcomes Research in Mental Health (LiCORMH)
Nepal	Expansion of existing R2HC-funded study in Haiti and Nepal, to rapidly adapt an existing intervention and apply it to earthquake affected areas in Kathmandu Valley.	Qualitative adaptation, mixed methods controlled cohort	Completed	University of Colorado, Transcultural Psychosocial Organization Nepal: (TPO Nepal)
Nepal	Evaluation of the cost-effectiveness and long-term impact of a combined nutrition/psychosocial intervention on the growth and development of children with Severe Acute Malnutrition (SAM) in the Saptari District of Nepal	Mixed methods, randomized controlled trial	Completed	Action Contre La Faim France, International Centre for Diarrhoeal Disease Research Bangladesh (ICDDR-B); District Public Health Office, Rajbiraj; Child Health Divison; NEEP
Pakistan	Evaluation of a multi-component behavioral intervention with conflict-affected adults (Problem Management Plus) (PM+)	Mixed methods, feasibility and fully powered cluster randomized trial	Completed	World Health Organization, Lady Reading Hospital, Peshawar; Human Development Research Foundation; Rawalpindi Medical College; University of New South Wales; Vrije Universiteit Amsterdam
South Sudan	Evaluation of a community-based program to protect children from developing epilepsy and improve the treatment and care of persons with epilepsy in onchocerciasis (‘river blindness’) endemic regions in South Sudan	Mixed methods and cohort studies (3 sites)	Ongoing	Amref Health Africa, Amref International University, Kenya; Ministry of Health, South Sudan; Global Health Institute, University of Antwerp, Belgium; University of Oxford, UK; Light for the World, Germany; OVCI la Nostra Famiglia, South Sudan; Mentor Initiative Sight Savers, South Sudan; and CUAMM, South Sudan
South Sudan	Evaluation of the impact of cash-based programming on intimate partner violence, including the potential role of mental health in this relationship	Mixed methods, controlled cohort	Ongoing	World Vision, Johns Hopkins University
Tanzania	Evaluation of a combined empowerment counseling and group psychotherapy intervention for female Congolese refugees who experienced intimate partner violence in the last year (Nguvu)	Mixed methods, feasibility cluster randomized controlled trial	Completed	Johns Hopkins University, Muhimbili University of Health and Allied Sciences, International Rescue Committee, the United Nations High Commissioner for Refugees, University of New South Wales
Tanzania	Evaluation of the feasibility and acceptability of a brief empowerment counseling intervention among pregnant women and girls with Congolese and Burundian refugees	Qualitative formative research, mixed methods cohort	Ongoing	World Health Organization, International Rescue Committee, Innovations for Poverty Action Tanzania, Global Women’s Institute, George Washington University
Uganda	Evaluation of a facilitated, group-based, guided self-help intervention with female South Sudanese refugees (Self Help Plus) (SH+)	Mixed methods, feasibility and fully powered cluster randomized controlled trial	Completed	World Health Organisation, HealthRight International; Makerere University; Johns Hopkins University; Institute of Psychiatry, Kings College London; University of New South Wales; United Nations High Commissioner for Refugees (UNHCR); University of Ottawa; University of Glasgow
Uganda	Adaptation and evaluation of a facilitated, group-based, guided self-help intervention with male South Sudanese refugees (SH+)	Qualitative adaptation, mixed methods feasibility and fully powered cluster randomized controlled trial	Ongoing	World Health Organization, Johns Hopkins University, HealthRight International, Ministry of Health Uganda, United Nations High Commissioner for Refugees
Uganda	Evaluation of enhanced child-friendly-space (CFS) interventions for children affected by conflict and displacement	Mixed methods, randomized controlled trial	Ongoing	World Vision and Columbia University

Six of the projects focus on innovations in the delivery of cognitive behavioral interventions, with several exploring new approaches to delivering evidence-based interventions. Bryant and coworkers are evaluating a new WHO transdiagnostic group intervention with young Syrian adolescents and caregivers in Jordan, delivered by lay workers [14]. El Chammay and colleagues are testing the feasibility and cost-effectiveness of a behavioral intervention delivered through an electronic (phone or web) application with Syrian refugees in Lebanon. Pluess and coworkers are evaluating the delivery of a transdiagnostic intervention delivered by phone with Syrian refugee children in Lebanon [15]. Rahman and colleagues have examined the cost-effectiveness of a multicomponent behavioral intervention delivered by lay helpers with conflict-affected adults in Pakistan [16]. Tol and colleagues have evaluated a guided self-help intervention with South Sudanese female refugees in northern Uganda [17–20], and are adapting and evaluating this intervention for use with male refugees.

Seven projects focus on multi-sectoral interventions, i.e. efforts to integrate MHPSS with activities in different sectors of humanitarian programming, including nutrition, gender-based violence, disaster risk reduction, and epilepsy. Bizouerne and coworkers tested the (cost) effectiveness of an intervention that combined nutrition and psychosocial support for young children with severe acute malnutrition in Nepal. Tol and colleagues studied an intervention that combined women’s intimate partner violence protection activities and cognitive processing therapy with Congolese refugee women in Tanzania [21, 22]. García-Moreno, Ellsberg and colleagues are evaluating the feasibility and acceptability of a brief empowerment counselling intervention in antenatal care for pregnant women and girl refugees from the Democratic Republic of the Congo (DRC) and Burundi in Tanzania who have experienced intimate partner violence. Welton-Mitchell, James and colleagues evaluated an intervention that combined disaster preparedness and psychological components in areas affected by earthquakes and floods in Haiti and Nepal [23, 24] (two projects). Jansen and colleagues are testing the effectiveness of a locally-developed community-based intervention with men in reducing gender-based violence in the eastern DRC who are perceived by their communities to be violent, and whether mental health conditions mediate or moderate in this process. Lako, Colebunders and colleagues will evaluate a community-based program in regions with onchocerciasis (river blindness) in South Sudan, aimed at protecting children from developing epilepsy and nodding syndrome and improving the care for people with epilepsy, including enhanced psychosocial support.

Three projects have focused on interventions widely implemented in humanitarian settings that have lacked research attention: psychological first aid [25] and child friendly spaces [26]. De Jong, Ager and coworkers conducted an evaluation of psychological first aid as applied in the Ebola crisis in Liberia and Sierra Leone [27]. Savage and colleagues evaluated child friendly spaces across crises in Jordan, Nepal and Uganda [28, 29], and are now conducting a trial of an enhanced CFS-design in Uganda.

One project focused on innovative methodologies to measure program impacts beyond self-reported data [30]. Panter-Brick and colleagues evaluated a brief psychosocial intervention delivered to Syrian refugee and Jordanian non-refugee adolescents, combining mental health self-reports [31], stress biomarkers [32, 33], and tablet-based cognitive testing [34].

One project does not involve specific MHPSS components, but is focused on the role mental health may play in moderating outcomes of poverty-reduction programming in humanitarian settings. Savage, Robinson, and colleagues are investigating whether mental health may be a significant variable with regard to the impacts of cash-based, food-security, programming on intimate partner violence in South Sudan.

## Initial findings

Thus far, results from seven projects have been finalized. Ager, Savage and colleagues conducted three quasi-experimental trials to evaluate the short- and long-term impacts of child friendly spaces (CFS) in Jordan, Nepal, and Uganda. CFS are a popular intervention aimed at increasing protection of children, improving psychosocial wellbeing, and mobilizing community resources. Findings showed variation in benefits across sites and outcomes. Analyses support earlier findings [28] of small to moderate impacts on psychosocial wellbeing indicators after participation in CFS [29]. However, with improved well-being amongst comparison populations over time, these intervention benefits were generally not evident at 1-year follow-up. There was wide variation in benefits across sites, outcomes and subgroups, but little evidence for impact on targeted community mobilization outcomes, findings which have shaped subsequent practice and guidance [26].

As part of De Jong, Ager and coworkers’ evaluation of psychological first aid training in the context of the Ebola crisis in Liberia and Sierra Leone, Horn and colleagues conducted a qualitative evaluation. The qualitative evaluation comprised semi-structured interviews with 24 trainers, 36 trainees, and 12 key informants. It found that psychological first aid (PFA) providers had a good understanding of active listening, but their responses to a person in distress were less consistent with PFA guidance. The authors warn of the myth of one-day training and urge for improved standardization of training for non-specialists [27]. A subsequent cluster randomized trial in post-Ebola Sierra Leone (*n* = 408) found that PFA-trained providers showed larger improvements than the control group on knowledge and understanding at 3- and 6-months follow-up, and better responses to a scenario at the 3-month follow-up. No differences were identified for professional attitude, confidence, and professional quality of life [35].

Panter-Brick and colleagues tested the psychosocial, physiological, and cognitive impacts of Advancing Adolescents, a program applying a profound stress attunement approach with war-affected youth, implemented by Mercy Corps as part of the No Lost Generation initiative in Jordan, Lebanon, Iraq, Syria and Turkey [30]. Youth in the randomized controlled trial showed small to moderate improvements in psychosocial wellbeing; notably, feelings of insecurity were alleviated up to one-year follow-up [31]. Hair cortisol concentrations dropped by one-third, demonstrating a beneficial regulation of physiological stress [32, 33]. However, no treatment effects were found for measures of cognitive function [34], or resilience [36], demonstrating that brief interventions can make notable impacts on psychosocial and biological stress, without necessarily changing broader social and developmental outcomes. These scientific findings informed programmatic decisions: Mercy Corps integrated elements of stress-attunement into its regional livelihood interventions and resilience-building efforts [30].

Rahman and colleagues evaluated the individual version of Problem Management Plus (PM+), a brief transdiagnostic intervention based on problem-solving and additional behavioral strategies delivered in 5 weekly 90-min individual sessions [37]. PM+ was tested in an individually randomized controlled trial in a conflict-affected, peri-urban setting in Peshawar, Pakistan [38]. PM+ was delivered by lay health workers in primary health care facilities with 346 adults screened for psychological distress. Results showed that three months after treatment the intervention group had significantly lower levels of psychological problems and functional impairment [39]. Further analyses found that PM+ was cost-effective [40]. In a separate study not funded by R2HC, PM+ was found to be effective with survivors of gender-based violence in informal settlements in Kenya [41], and has since been made available by WHO as an open access resource (https://www.who.int/mental_health/emergencies/problem_management_plus/en/). PM+ is now available in 13 languages, has been the subject of research in various populations (including research focused on scaling-up) [42, 43], and is being used by > 10 humanitarian agencies [44]. A group version has also been made available (https://www.who.int/publications/i/item/9789240008106) [45, 46].

Welton-Mitchell, James, and their team’s project builds on the observation that many people do not engage in even low-cost disaster preparedness, such as making a disaster supply kit, putting important documents in a safe place, securing dwellings and furniture, and discussing family evacuation plans. This may be partly due to mental health difficulties, including those associated with prior disaster exposure. With national partners, and with input from local clinicians and community members, they developed and tested a culturally-adapted, hybrid mental health and disaster preparedness 3-day manualized group intervention. Two randomized controlled trials in flood-affected communities in Nepal and Haiti and one matched cluster comparison in earthquake-affected communities in Nepal were conducted with a total of 1200 community members. Results across studies indicate that intervention participation was associated with increased disaster preparedness and social cohesion. Decreased mental health symptoms were also observed in two of the three studies. This study shows that attention to psychosocial components may make disaster preparedness more effective, and likewise, attention to preparedness may improve wellbeing [23, 24, 47]. The interventions developed and tested in Haiti and Nepal have been used when responding to new disasters.

Tol, Van Ommeren and colleagues evaluated the benefits of a group-based, facilitated, guided self-help intervention in reducing psychological distress of female South Sudanese refugees living in settlements in northern Uganda. The intervention was developed by WHO and is based on acceptance and commitment therapy, a modern form of cognitive behavioral therapy that includes mindfulness-based components. The 5-session intervention is delivered through audio-recorded materials and a self-help book in workshops of 20–30 people by briefly trained lay facilitators [48]. The intervention was adapted and piloted with both men and women, which found further adaptation was required for male refugees [18, 19]. A subsequent cluster randomized trial with female refugees in 14 villages (*n* = 694) found benefits at the 3-month follow-up with regard to psychological distress, depressive and posttraumatic stress symptoms, feelings of anger, functional impairment, and subjective wellbeing [20]. Further adaptation and evaluation with male refugees is currently ongoing, and the intervention is evaluated as a prevention intervention with refugees in various European countries (http://re-defineproject.eu/).

Finally, Tol and colleagues developed an 8-session group intervention combining a women’s protection intervention (empowerment counseling, including safety planning, a danger assessment, and provision of information on protection options) and a psychological intervention (group cognitive processing therapy), aimed at reducing intimate partner violence victimization and psychological distress for female Congolese refugees in Tanzania who experienced intimate partner violence in the last year [22, 49, 50]. A feasibility cluster randomized controlled trial was conducted with *n* = 311, results of which are currently under review for publication. A similar effort at integration is currently ongoing with a different humanitarian organization in a project with displaced populations in Ecuador and Panama.

## Implications for research-practice

The initial findings from this set of studies - and preliminary response to them by humanitarian agencies - illustrate two related issues. The findings clearly confirm the critical role that research can play in informing humanitarian MHPSS practice, implying that more such research should be pursued. However, the findings also bring into focus potential risks of expanding research on humanitarian MHPSS in the absence of concerted efforts to simultaneously strengthen relationships between scholars and practitioners.

The research findings regarding CFS may illustrate these related issues. The initial findings of CFS research highlight how widely-shared assumptions (e.g., CFS are a key way to improve child protection outcomes, and mobilize communities in support of children) may not be confirmed in controlled studies in all settings. Such knowledge is clearly informative for humanitarian decision making, and highlights the potential role that research can play. Yet, it is important that such findings are interpreted with caution and shared and discussed widely with those delivering humanitarian programs. Seemingly based on the initial findings reported above some agencies are now encouraging a move away from CFS, illustrating that decision making following the generation of evidence requires continued partnerships between researchers and practitioners. A decision to completely move away from CFS in our opinion is too hasty. Supported by evidence of observed, yet varied, benefits of CFS across different implementation contexts, improving existing practices may, instead, prove a more appropriate strategy [26], alongside continued research. Opportunities for improvements to CFS programming may lie both in bridging the critical ‘quality gap’ and in the enhanced contextualization of CFS practices to local culture and context.

The observation that new research findings may result in boom-and-bust decisions for specific types of humanitarian interventions highlights the need for sustained scholar-practitioner interactions once a research study is concluded and the findings and implications are being interpreted and considered for use. Similarly, continued interactions are necessary to ensure that other interventions that are widely implemented in practice are rigorously evaluated. The importance of continued interaction between researchers and practitioners may be particularly urgent in the humanitarian space, where there is a strongly felt need for clear cut answers and simple, readily deployable and scalable solutions. Such interaction must engage the processes and people involved in decisions and policy making - not just the technical experts. For humanitarian practice and policy to improve through research, we believe enhanced efforts at communication and engagement are needed from both scholars and practitioners. For academic researchers, we believe it is important that the selection of research topics more closely aligns with the needs of humanitarian practitioners on the ground. A previous research priority setting initiative that involved practitioners highlights this point. In this initiative, the most highly prioritized research questions were different from the issues most heavily discussed in the academic literature, such as the exact prevalence of PTSD symptoms in populations affected by humanitarian crises. In fact, the most highly prioritized research questions were more applied research questions, such as: optimal methods to conduct needs assessments; indicators for monitoring and evaluation; and improved understanding of MHPSS needs and interventions from the affected populations’ point of view. Also, we believe it is critical that researchers need to do better in ensuring their outputs reach practitioners and policy makers in accessible formats and timely ways. For humanitarian practitioners, we believe there is a need for improved capacity building to appropriately build on evidence across the project cycle: from structured needs assessments; selection of evidence-informed interventions and developing strong theories of change; to designing and drawing conclusions from programmatic monitoring and evaluation efforts. To reduce tensions between humanitarian practitioners and researchers, we believe it is important that expectations concerning and strategies to achieve research impact are openly discussed at the outset, at all stages of research projects, and following the generation of evidence.

A second observation we draw from the initial findings concerns implementation of evidence-based interventions. Where the positive benefits of interventions have been identified, further effort is required to ensure that interventions are actually used in routine programming settings [51], given the observation that MHPSS interventions that have shown to be effective are not widely implemented in humanitarian settings [9]. This research-to-implementation gap may be due to design challenges (e.g., current evidence-based interventions address mental health conditions that are not the primary concern of humanitarian responders, require resources that humanitarian agencies do not have, or were designed with little sense of ownership by humanitarian agencies) or due to implementation challenges (e.g., current evidence-based interventions are challenging to implement with fidelity to intervention manuals in resource-poor humanitarian settings). Real-world delivery of evidence-based interventions can be improved by addressing both design and implementation considerations along the path from research-to-practice in a collaborative manner between researchers and practitioners [52].

With regard to design, further work on developing interventions that from the start stand a chance of being used in highly resource constrained and damaged health systems is likely helpful. Previous research on knowledge translation in other fields has shown that the chances for long-term adoption of interventions after their testing in studies are improved if interventions were co-created with end-users [53] – while respecting questions of power and influence [52]. Such approaches would require better alignment and collaboration with large scale funding and operations, seeing agencies and donors as the end-users, to integrate rigorous research designs at scale and funding for them into programming. They also need to build on existing local solutions and human resources, thus integrating historical and long-term thinking which leads to a better local absorption of novel interventions.

With regard to implementation, scholars, practitioners and funders may fruitfully collaborate on “implementation research”, aimed at informing how implementation of existing evidence-based interventions may be optimized in real-world settings [54]. For example, a number of currently ongoing R2HC-funded studies are focused on identifying feasible delivery of evidence-based psychological interventions through different types of methods, including telephone and mobile application-based delivery. All these areas would benefit from close, equitable and sustained dialogue between researchers and practitioners, to marry excellence and relevance in the implementation of robust evidence for lasting benefits to crisis-affected populations. Further recommendations on how to ensure fruitful collaborations between researchers and humanitarian agencies have been published by the Nuffield Council on Bioethics [52].

## Remaining knowledge gaps

In reviewing the current R2HC-funded MHPSS research portfolio, four specific knowledge gaps may be noted. First, important questions remain regarding how evidence-based interventions tested in humanitarian contexts can be scaled up. For example, what role can new technologies play in facilitating the transition from controlled evaluation to scale-up? What kind of sector-specific, organizational-level, dynamics may facilitate or impede the integration of evidence-based MHPSS interventions into different types of humanitarian programming (e.g., violence prevention, strengthening livelihoods, preparedness and other forms of disaster risk reduction)? What are the optimal care platforms in which interventions can be integrated (e.g. collaborative care and stepped care models)? What kind of minimum training, supervision, and referral mechanisms need to be in place to scale-up responsibly and safely? How can we consistently adhere to best practice guidelines for adaptation of interventions to specific cultural contexts? What kind of partnerships (with academic researchers, humanitarian organizations, funders, the media, and local communities) are required to generate credible evidence, establish productive dialogue, and improve scientific uptake? In starting to answer these questions, recent research has built on participatory Theory of Change methodology to support the development of scale-up strategies [55].

Second, we need to understand how to address the needs of under-researched populations and mental health conditions - reflecting gaps in contemporary research on MHPSS more broadly. For example, none of the research projects focused specifically on the elderly, or other marginalized groups, e.g., sexual minorities, children with developmental disorders, or individuals with disabilities. Similarly, research focused specifically on men affected by humanitarian crises is less common. Moreover, no studies have targeted severe mental disorders (e.g., psychosis or bipolar disorder), suicide prevention, or alcohol and drug misuse interventions, even though these are critical but under-researched concerns in humanitarian settings [56–58] and guidelines have been published focused on providing services for these concerns in non-specialized humanitarian health care systems (e.g., primary care) [59].

Third, there remains a knowledge gap regarding how to effectively build on local existing supports, such as religious and traditional healing and community-level social support systems, but also including professionally high-level functioning local NGOs, research teams and governing bodies. Most of the studies in the R2HC MHPSS portfolio have pragmatically adapted and evaluated interventions developed outside of the context in which they are applied. There is a tension between the need for interventions that can be rapidly adapted and deployed in new humanitarian crises, and the preference to build humanitarian programming on locally available resources that support mental health and psychosocial wellbeing. More research is needed that assesses the effectiveness of locally available and used supports, and the best processes to engage with these supports [60]. For example, the studies aimed at integrating mental health considerations in disaster preparedness in Haiti and Nepal build on local support practices by: encouraging community members to provide peer support to neighbors with mental health concerns; encouraging mental health-specific help-seeking with both informal and formal support networks; and recognizing the role of culturally-specific beliefs and practices. Similarly, the work of Living Peace, a local non-governmental organization working to reduce gender-based violence in Eastern DRC is collaborating with scholars from neighboring Rwanda. The Living Peace intervention works with community volunteers who are trained to guide groups of (perceived to be) violent men through 15-week group sessions attended by 15 men. The project builds on locally existing solutions and aims to evaluate its impact to identify strengths and weaknesses that can help to further improve the intervention. In both the disaster preparedness and Living Peace research projects (as well as several others), the development of initial research partnerships was supported by seed funding, so that the initial research questions were jointly developed. In Haiti, for example, the intervention curriculum built on an earlier intervention which was jointly developed with survivors of the 2010 earthquake, and included coping mechanisms drawing on local belief systems, stories, songs, dance and humor.

There are several potential barriers to conducting controlled evaluations of locally available supports, which will require careful consideration. Some of these are related to differences in theories of change between external researchers and local practitioners, and will involve questions of (epistemic) decision making. E.g., shamanistic healing practices may be perceived as primarily aimed at thwarting the influence of witchcraft, rather than a reduction on a specific set of emotional difficulties. Certain healing systems may also have strict rules around concealing effective ingredients of interventions, and interventions may not be easy to deliver in a structured manner. Barriers may also be related to current technical limitations, e.g. a lack of reliable and valid outcome instruments to assess changes brought about through local practices, or a lack of the appreciation of the dynamic nature of local healing practices [61]. Nevertheless, systematic reviews of quantitative studies have found that traditional healing seems effective in relieving psychological distress [60].

Fourth, more needs to be learned about how to ensure the quality of evidence-based interventions when implemented in real-world settings. The R2HC MHPSS portfolio has focused largely on (randomized) controlled trials. These trials are pragmatic trials, implemented in real-world humanitarian settings broadly representative of the settings in which humanitarian crises occur (i.e., they more closely resemble effectiveness than efficacy trials). However, such trials often have at their disposal resources to ensure implementation quality that are not commonly available to general humanitarian practitioners (e.g., in terms of training, supervision and implementation quality management). Future studies should therefore focus on testing interventions with quality management scenarios that are more typical for humanitarian agencies. Some of the completed studies are resulting in useful tools that can be used for quality management in real-world contexts. For example, WHO is developing a psychological interventions operational manual, including guidance on selection, adaptation, and monitoring and evaluation of interventions.

## Conclusions

In conclusion, the R2HC MHPSS portfolio is starting to contribute to answering essential questions regarding the effectiveness of a range of MHPSS interventions in humanitarian settings – a field where research and practice have historically been misaligned. While critical knowledge gaps remain, the initial findings illustrate both the importance of research for humanitarian decision making (e.g., because research is not confirming widely held assumptions about the effectiveness of popular MHPSS interventions to achieve intended outcomes), and the need for longer term partnerships between researchers and practitioners to bring research into practice – and practice into research (e.g., to ensure appropriate humanitarian decision-making based on generated evidence, and the implementation of evidence-informed interventions in humanitarian practice). Bridging the gap between MHPSS research and practice will require compromise and efforts from both researchers and practitioners.

Key remaining knowledge gaps include questions around how to: scale up MHPSS interventions that have shown to be effective in humanitarian settings; address the needs of under-researched populations and mental health conditions; build on local existing supports; and ensure quality of MHPSS interventions as they move from controlled research studies to the real-world.

## Data Availability

Information about studies described here can be found at: https://www.elrha.org/programme/research-for-health-in-humanitarian-crises/
